# A Patient-Centered Asthma Management Communication Intervention for Rural Latino Children: Protocol for a Waiting-List Randomized Controlled Trial

**DOI:** 10.2196/18977

**Published:** 2020-12-01

**Authors:** Robin M Dawson, Sue P Heiney, DeAnne Hilfinger Messias, Dennis Ownby

**Affiliations:** 1 College of Nursing University of South Carolina Columbia, SC United States; 2 Augusta University Augusta, GA United States

**Keywords:** asthma, mHealth, mobile app, Latino, pediatrics, family-centered care, school nursing, rural health, RCT, mobile phone

## Abstract

**Background:**

Rural Latino children with asthma suffer high rates of uncontrolled asthma symptoms, emergency department visits, and repeat hospitalizations. This vulnerable population must negotiate micro- and macrolevel challenges that impact asthma management, including language barriers, primary care access, parental time off from work, insurance coverage, distance from specialty sites, and documentation status. There are few proven interventions that address asthma management embedded within this unique context.

**Objective:**

Using a bio-ecological approach, we will determine the feasibility of a patient-centered collaborative program between rural Latino children with asthma and their families, school-based nursing programs, and primary care providers, facilitated by the use of a smartphone-based mobile app with a Spanish-language interface. We hypothesize that improving communication through a collaborative, patient-centered intervention will improve asthma management, empower the patient and family, decrease outcome disparities, and decrease direct and indirect costs.

**Methods:**

The specific aims of this study include the following: (1) Aim 1: produce and validate a Spanish translation of an existing asthma management app and evaluate its usability with Latino parents of children with asthma, (2) Aim 2: develop and evaluate a triadic, patient-centered asthma intervention preliminary protocol, facilitated by the bilingual mobile app validated in Aim 1, and (3) Aim 3: investigate the feasibility of the patient-centered asthma intervention from Aim 2 using a waiting-list randomized controlled trial (RCT) to investigate the effects of the intervention on school days missed and medication adherence.

**Results:**

Mobile app translation, initial usability testing, and app software refinement were completed in 2019. Analysis is in progress. Preliminary protocol testing is underway; we anticipate that the waiting-list RCT, using the refined protocol developed in Aim 2, will commence in fall 2020.

**Conclusions:**

Tailored, technology-based solutions have the potential to successfully address issues affecting asthma management, including communication barriers, accessibility issues, medication adherence, and suboptimal technological interventions.

**Trial Registration:**

ClinicalTrials.gov NCT04633018; https://www.clinicaltrials.gov/ct2/show/NCT04633018

**International Registered Report Identifier (IRRID):**

DERR1-10.2196/18977

## Introduction

### Background

Rural Latino children with asthma and their families may experience unique, multilevel barriers to effective asthma management, contributing to disparate health outcomes and broader health disparities. Children with asthma insured by Medicaid disproportionately represent emergency department (ED) visits, including repeat visits [1], and are more likely to require repeat hospitalizations for uncontrolled asthma symptoms [2]. Patients and families who are able to address environmental triggers, address medication adherence, and access timely primary care visits have fewer exacerbations requiring emergency interventions, resulting in significantly reduced direct and indirect costs, fewer lost school days and workdays, and improved quality of life. Evidence suggests that Latino people are more likely to access and use mobile health (mHealth) apps, especially if the app contains a Spanish-language interface [3]; however, there are no asthma management apps currently available in Spanish.

### Barriers and Solutions to Effective Asthma Management

#### Communication With and Between Health Care Providers

Sporadic availability of language interpreters, especially in rural areas and in systems with limited resources, often results in providers “getting by” with problematic solutions, such as child interpreters or overestimating their ability to communicate in the target language [4]. Beyond the obvious communication challenges that participants in language discordant interactions can encounter, parents report significant issues in conflicting management styles and communication between health care providers [5], causing parents to become frustrated with their child’s care [6].

#### Continuity of Care

Parents express a preference for primary care management of their child’s asthma, and patients who have primary care appointments soon after an asthma exacerbation requiring an ED visit are less likely to require a repeat ED visit during the following month [7]. However, there are real and perceived barriers experienced by these families, including the inability to schedule urgent visits or parental work demands [5]. Further, continuity of care and primary care access may be compromised due to insurance status [8]. The vast majority of patients do not follow up after suffering an asthma exacerbation requiring emergency intervention, even when they have insurance and an identified primary care provider (PCP) [9].

#### Lack of Adherence to Medication

Latino patients are less likely to take asthma medications as prescribed when compared to non-Latino White people [10]. Cost, access challenges, and knowledge deficits contribute to suboptimal medication adherence, often related to difficulty obtaining the appropriate medications and devices, such as spacers or nebulizer machines, due to lack of insurance coverage or cost. Knowledge deficits (eg, parental beliefs that prolonged use of asthma medication can cause weakening of the lungs, bone density loss, medication dependence, or cardiac complications) and parental perceptions regarding the necessity of their child’s asthma medication can negatively influence preventative therapy adherence [11].

#### Fragmented Technological Interventions

Technological interventions, including mobile apps to assist the patient with asthma management, are readily available for use on smartphones. However, few apps incorporate functions associated with improved patient outcomes [12]. For example, some asthma apps offer the ability to track medications and asthma symptoms but do not allow the correlation and sharing of data. There are no apps available currently that (1) allow secure storage and correlation of medication administration, symptoms, and exacerbations requiring medical intervention, (2) allow health care providers to access the patient data to examine trends over time, and (3) are available in Spanish.

#### School Nursing Programs

School nursing programs have the potential to positively impact childhood chronic disease management, including asthma, as approximately 90% of elementary and secondary school–age children are enrolled in public schools in the United States [13]. School-based educational programs have been shown to increase parents’ knowledge regarding environmental triggers for their children’s asthma [14,15], but school-based services often do not meet National Heart, Lung, and Blood Institute asthma management guidelines [16]. In addition, while educational programs delivered by school nurses are effective in improving patient asthma knowledge, these nurses face their own unique barriers, including time to attend educational programs and provide case management for the students with asthma, specific knowledge deficits, and having confidence in their ability to independently implement asthma programs [17]. The challenges school nurses face in caring for their students with asthma are compounded when parents have limited English proficiency. However, a clear understanding of how existing relationships and systems can be leveraged and coordinated has the potential to streamline the disease management process and effectively address outcome disparities among rural Latino children with asthma.

### Contribution of Proposed Project to Advancing Scientific Knowledge and Public Health

#### Overview

The proposed feasibility study is supported by a K23 award; this type of award provides individuals who have a clinical doctoral degree with an intensive, supervised, patient-oriented research experience. This study addresses a gap in our current understanding of best practices for asthma management in vulnerable populations. While the majority of the 56 million US Latino people live in urban areas, this population is increasingly moving to nontraditional, rural settlement areas such as South Carolina. Latino children born in rural areas are significantly more likely to be poor, have limited access to services, and live in substandard housing associated with migrant farming [18]. My previous research has identified unique barriers impacting asthma management in rural Latino children, including parental documentation status, access to transportation, and distance to specialty sites [19]. My focus on a targeted, patient-controlled intervention taking multilevel pressures into account may reduce the disparities suffered by this vulnerable population.

#### Innovation

Innovative aspects of this project include the bio-social-ecological framework, the patient-centered and collaborative approach, and the utilization of bilingual technology. The bio-ecological perspective guiding the research addresses the *specific challenges rural Latino children and their families face* when managing asthma care embedded in a complex interaction of environments, agencies, and systems. The research incorporates patient-centered and community-based collaboration, building on a patient- and family-directed collaboration, rather than an institutionally situated intervention, and leveraging existing systems such as school nursing programs. Bilingual technology addresses language discordance. The app does not translate; rather, the parent and caregiver interface is in Spanish and input is securely transmitted to the school nurse and accessible in English. The use of the Spanish-language version of *AsthmaMD* (AMD-Sp), a free asthma management app, will facilitate day-to-day communication between the school nurse and the parent or caregiver, minimizing the daily need for an interpreter.

### Theoretical Framework

My preliminary studies and proposed study are guided by a bio-ecological conceptualization of the complex interactions and intersections of multilevel factors with childhood asthma management in a specific vulnerable population group: rural Latino children with asthma. I will use Bronfenbrenner’s Process-Person-Context-Time model to organize the study approach, intervention design, and data analysis (see Table 1) [20]. The concepts in this model allow for identification and examination of relevant variables that contribute to poorer health care outcomes (eg, communication issues within and between patients and system representatives, where people live, health care accessibility, socioeconomic status, demographics, individual knowledge, and how people and environments change over time).

**Table 1:** Bronfenbrenner’s Process-Person-Context-Time model and ecological levels.

Ecological level	Associated factors
Microsystem	Patient: knowledge deficits and medication adherence School nurse: knowledge and informational deficits Primary care provider: knowledge and informational deficits
Mesosystem	Interactions between the patient and family and school nurses Interactions between the patient and family and provider Interactions between the provider and school nurses
Exosystem	Effects of transportation issues Effects of work attendance constraints Effects of documentation status
Chronosystem	Knowledge change over time Medication adherence over time Biophysical measures over time Emergency department visits (retrospective and prospective) Sustainability of program after feasibility study is complete

### Preliminary Studies

The first preliminary study was *Patterns of communication technology utilization for health information among Hispanics in South Carolina: Implications for health equity* [19]*.* This cross-sectional, descriptive study examined patterns of technology use and health care information seeking and service access among South Carolina–based Latino people with limited English proficiency. Data were collected in two waves; 361 Latino people completed surveys. Self-reported accessibility and utilization of cell phones increased (89% in 2011 to 96.6% in 2015-2016), supporting the feasibility of using a technology-based approach in this proposal.

The second preliminary study was *Exploring app features with outcomes in mHealth studies involving chronic respiratory diseases, diabetes, and hypertension: A targeted exploration of the literature* [21]*.* This review identified 27 studies that utilized mobile apps in the management of selected chronic diseases: diabetes, hypertension, and asthma. The use of at least one of four specific app features was associated with improved patient outcomes; the app proposed for the study, *AsthmaMD*, contains two of these features, including clinical data shared with health care providers through interoperability and incorporation of an evidence-based clinical decision support system.

The third preliminary study was *Urban-rural differences in school nurses’ asthma training needs and access to asthma resources* [22]. This survey study was conducted with school nurses in South Carolina, including those in the Lancaster County School District (LCSD), which had 100% participation rate. At the time, only 16% of participants were implementing an asthma program in their school, yet the vast majority (87%) of nurses expressed a desire for further asthma training programs.

The preliminary data from these studies substantiate the uptake of technology by rural Latino people, as seen by improved patient compliance with certain features of apps that are contained in the *AsthmaMD* app and an increase in school nurses’ willingness to participate in the study.

## Methods

### Study Overview

This project received funding from the National Heart, Lung, and Blood Institute of the National Institutes of Health (NIH) (2017-2022). Guided by a bio-ecological theoretical framework, the primary goal of this research is to develop and evaluate a patient-centered collaborative intervention between rural Latino children with asthma and their families, school-based nursing programs, and PCPs, facilitated by the use of a smartphone-based Spanish-language mobile app. The three specific aims are as follows:

1. Produce and validate a Spanish translation of an existing asthma management app and evaluate its usability with Latino parents of children with asthma.
2. Develop and evaluate a triadic, patient-centered asthma intervention preliminary protocol, facilitated by the bilingual mobile app validated in Aim 1.
3. Investigate the
  feasibility
  of the patient-centered asthma intervention from Aim 2 using a waiting-list randomized controlled trial (RCT) to investigate the effects of the intervention on school days missed and medication adherence.

### Aim 1: Produce and Validate a Spanish Translation of the App

#### Overview

**Figure 1:**
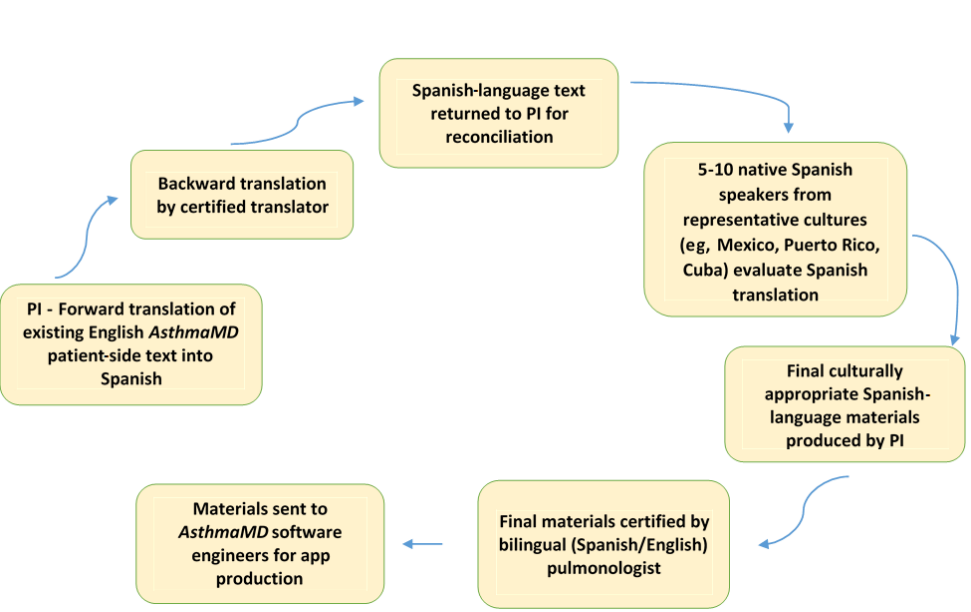
Systematic translation process of an existing asthma management app. PI: principal investigator.

Figure 1 shows the systematic translation process of an existing asthma management app, *AsthmaMD*. We will create a Spanish-language version of *AsthmaMD*, AMD-Sp, a free asthma management app, and will evaluate its usability with Latino parents of children with asthma. Users of the existing *AsthmaMD* app are able to (1) track their maintenance and rescue medications as well as set medication administration reminder alerts, (2) produce an asthma action plan, (3) note asthma symptoms and exacerbations, including ED visits, and (4) share this information with health care providers securely. An unpublished analysis by *AsthmaMD* developers demonstrated that English-speaking adult users with higher app engagement had improvement in their pulmonary function measurements by a minimum of 10% after 6 months of usage*.* Three independent certified translators will render the English app text into Latin American Spanish, as the population targeted in this study is primarily of Mexican origin, using methods adapted from Squires et al [23] (see Figure 1). *AsthmaMD* software engineers will use the certified, translated materials to produce AMD-Sp for the iOS platform. The state of South Carolina has a contract with Verizon Wireless that will allow me to minimize the costs associated with the hardware (ie, iPhones) and data plans necessary to implement the intervention.

#### Participant Recruitment

Once developed, I will recruit a convenience sample of 5 limited English–proficient Latino parents or caregivers of children with asthma to use the app for a week. They will examine the app for learnability, memorability, number of errors made in use, and satisfaction [24]. I will employ a usability heuristic to identify potential problems [25]. Participants will receive a US $25 cash incentive on return of the phone.

#### App Refinement

We will consider modification of app software as needed at each step of the evaluation, including translation, usability, and protocol development.

### Aim 2: Develop and Evaluate the Preliminary Protocol for a Triadic, Patient-Centered Asthma Intervention

#### Overview

**Figure 2:**
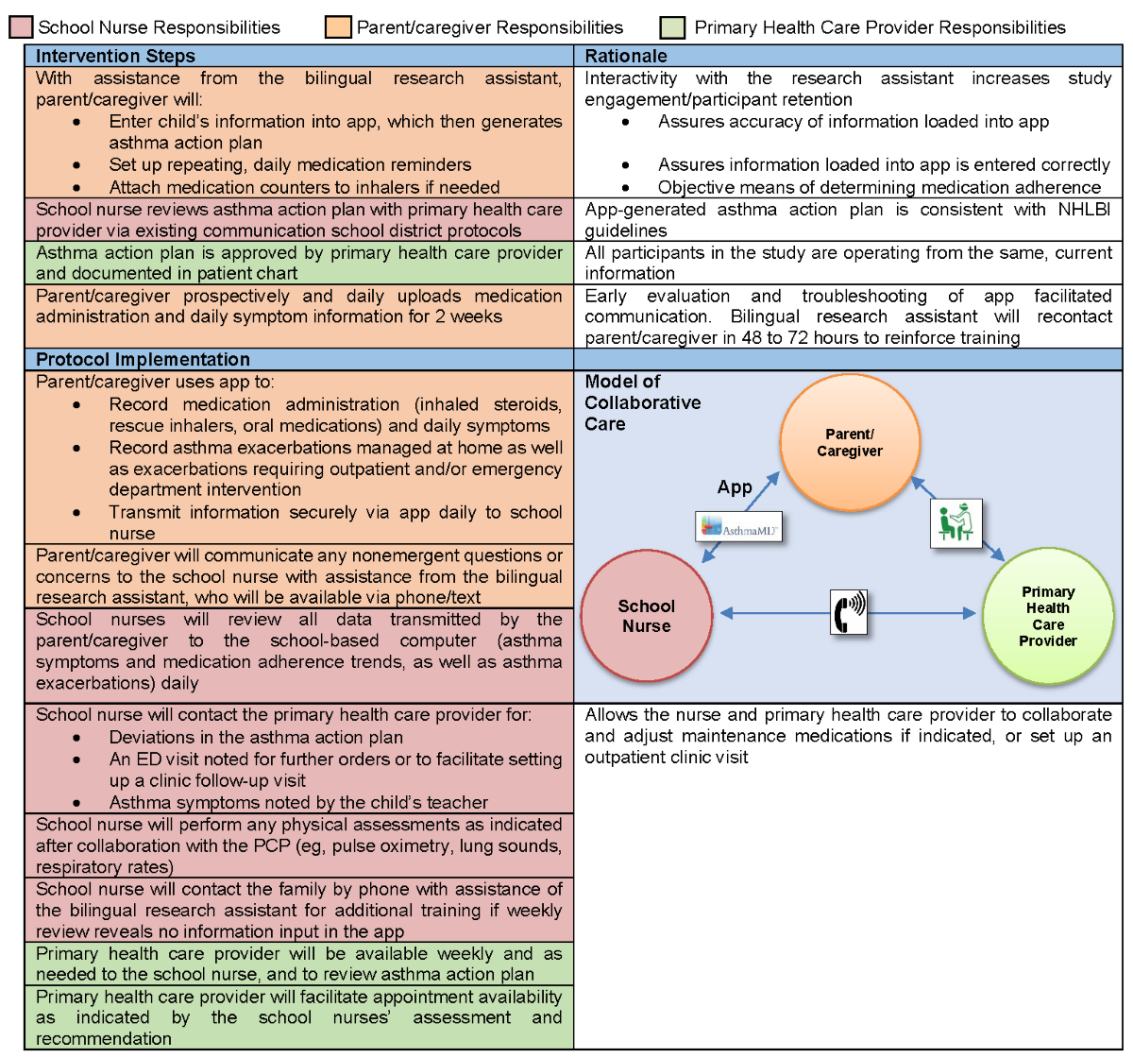
Preliminary protocol. ED: emergency department; NHLBI: National Heart, Lung, and Blood Institute; PCP: primary care provider.

The goal of this aim is to develop a pragmatic, acceptable, and financially sustainable *model of collaborative care* intervention (see Figure 2) facilitated by the Spanish-language mobile app validated in Aim 1. I will utilize the data from my study *Experiences of rural Southeastern Latino parents of children with asthma* to identify patient and family issues of importance to this collaboration. To situate the collaboration, I will utilize the analysis of relevant South Carolina epidemiological data to account for macrolevel issues that may impact the collaboration. Once developed, evaluated, and refined, the protocol generated by Aim 2 will be used in Aim 3.

#### Participant Recruitment

##### Overview

Five triads of participants will participate in the preliminary protocol. First, I will arrange a meeting with clinic and school representatives to develop an acceptable communication protocol between these institutions. As a former full-time, and now part-time, employee I have an established relationship with the largest pediatric primary care facility in Lancaster, South Carolina, which has a large Latino patient base, as well as with the LCSD. The director of student services, along with the director of nursing services, has already made a commitment to this study. Participants include three distinct groups, listed in order of recruitment: (1) a PCP, (2) Latino children with asthma and their parents or caregivers, and (3) school nurses. SPH and DHM will guide me in subject recruitment [26] as discussed in the following sections.

##### Primary Care Provider

A local pediatrician who has practiced primary care pediatrics in Lancaster County for over 15 years has agreed to participate; her consent will be obtained prior to initiating the identification of potential Latino children participants.

##### Latino Children With Asthma and Their Parents or Caregivers

I will purposively recruit a convenience sample of 5 Latino children with asthma, along with their parents or primary caregivers who have limited English proficiency; who reside in Lancaster County, South Carolina; and who are patients at the local pediatrician’s clinic. The pediatrician will assist in the identification of appropriate participants, and I will also utilize Spanish-language recruitment flyers to be placed at the outpatient clinic. Once participants are identified and express interest in participating in the study, a bilingual research assistant will contact them personally to give an overview of the study and obtain consent (parents) and assent (child), as well as appropriate Health Insurance Portability and Accountability Act (HIPAA) and medical records release forms. Inclusion criteria are as follows: (1) children who self-identify or family-identify as Hispanic or Latino, (2) school-aged (5-12 years) children who attend school within the LCSD, (3) children who have received a diagnosis of asthma from a health care provider and are taking a controller medication, and (4) parents’ or primary caregivers’ (eg, grandparents and extended family) language of preference is Spanish.

##### School Nurses

The LCSD includes 21 schools serving 11,500 students. Each of these schools has at least one dedicated nurse: a licensed practical nurse or a registered nurse. Once patient participants are identified, I will contact the nurses at the school they attend to review the study and obtain their consent to participate. Once the participants have been enrolled, they will participate in targeted training, as described in the following sections.

#### Training

##### Primary Care Provider and School Nurses

I will conduct one-on-one sessions with the PCP and school nurses to explain how the patient enters information (ie, medication administration and symptoms) into the app. For the school nurses, I will demonstrate how to access the digitally encrypted information using their site-based computers, which are available at all LCSD schools, as well as provide a short manual of instructions that will include screenshots and frequently asked questions.

##### Parents and Caregivers of Latino Children With Asthma

An iPhone 5 will be provided for each participant, to be returned at the conclusion of the study. The phone will have AMD-Sp installed. They will review the information on the phone data plan, how to use the iPhone, and how to enter information into the app. At the completion of Aim 2, parents and guardians will receive US $100 cash per family.

#### Intervention: Developing a Model of Collaborative Care

##### Overview

The intervention development stage will begin after completion of the initial educational activities. Enrolled participants will be followed over 3 months using the preliminary protocol (see Figure 2), with the understanding that this protocol is adaptable and flexible as the study progresses.

##### Parents and Caregivers of Latino Children With Asthma

As each subject is recruited and enrolled, a bilingual research assistant will conduct a training session with the child as well as with the parent or caregiver in Spanish. They will also be given a short instruction manual, in Spanish, with screenshots and frequently asked questions.

#### Data Collection, Analysis, and Protocol Refinement

##### Formative Data Collection for All Participants

Demographic information will be obtained from all enrolled participants: school nurses, parents and caregivers, children, and health care providers.

##### Quantitative Data Collection

We will perform medication counts monthly; asthma medications that do not have counters or individual vials, such as some metered-dose inhalers, will be fitted with a PuffMinder, a digital inhaler dose counter. These results will be compared to the self-report medication adherence information entered into the app by the parent or caregiver.

##### Qualitative Data Collection

Postintervention data regarding the experiences of the participants in this collaborative intervention will be obtained through a postintervention focus group facilitated by the principal investigator (PI) with the PCP and school nurses; in addition, five interviews will be conducted by a bilingual research assistant with the Latino families. These focused, semistructured encounters will be guided by open-ended questions detailed in interview guides to be developed. Audio data will be digitally recorded, then transcribed in Spanish by Verbal Ink; data will be managed with the NVivo 10 platform (QSR International) [27]. A qualitative, descriptive, thematic analysis approach will inform the transcript analysis. The data analysis will be conducted by the PI with input from DHM, who has an extensive background in qualitative research. She and I will be responsible for the iterative process of data analysis, and will conduct open and focused coding as well as thematic analysis [28].

### Potential Limitations and Solutions

The results from Aims 1 and 2 will be used to refine the intervention for Aim 3. For example, if we find that the PCP prefers to communicate with the school nurses via secure email instead of by phone, we will adjust the procedures. If the school nurses have issues accessing the data, I can offer additional training. As self-reports of adherence may be inflated, we will compare medication counts with information entered into the app by the parent or caregiver. Finally, even though AMD-Sp is easy to use and will require approximately five minutes of the parent’s or caregiver’s time to log symptoms and medication adherence information, if the parent or caregiver is having difficulty using the technology, my bilingual research assistant will provide additional support. I will be tracking app usage for the first week of each new enrollee to discuss concerns and strategies. We are aware of the possibility that these participants may have some unknown difficulties. If this preliminary step reveals this to be an issue, we can consider simple modifications to the study plan, such as the use of a short messaging service (ie, text messaging, which is associated with improved chronic disease management) in lieu of the app.

### Aim 3: Investigate the Feasibility of the Patient-Centered Asthma Intervention

#### Overview

With the results from Aim 2, I will refine the collaborative intervention and produce the protocol to be utilized in Aim 3. Using a waiting-list RCT, I will then evaluate the feasibility and acceptability of the intervention, including dosing, fidelity, recruitment, and retention [29], and investigate the effects of the intervention on school days missed and medication adherence.

#### Participant Recruitment

Participant recruitment will occur on a rolling basis and will follow the same procedure as noted in Aim 2, except that 20 Latino families will be recruited, following the same inclusion and exclusion criteria, for this arm of the study. Participant responsibilities will also remain as noted in Aim 2, unless there is a need to modify them based on the qualitative results from Aim 2. As the families are enrolled in the study, they will be randomly assigned to either the intervention group or the waiting-list control group. After the initial 10 intervention group participants complete the 6-month data collection period, the 10 waiting-list control group participants will then proceed with the 6-month intervention. In addition to Dr Ambati, there are additional pediatricians and nurse practitioners at the primary care clinic site, as well as other pediatric providers in the area served by the LCSD. An invitation to participate will be extended to them as well. Participant training will follow the procedures outlined previously. At the completion of the study, parents and guardians will receive US $100 cash per family.

#### Intervention

The refined protocol developed at the end of Aim 2 will be used to direct the intervention stage of Aim 3; it is anticipated that the steps will be similar. Participants will be followed for 6 months.

#### Data Collection

Anticipated quantitative data that will result from Aim 3 include the information entered into the app by the parent or caregiver, which are then encrypted and transmitted to the school nurse. These data will include selected NIH and Agency for Healthcare Research and Quality standardized asthma outcomes [30], including the primary outcome measures of medication adherence information (ie, asthma medication ratio [31], which is predictive of childhood asthma ED visits and hospitalizations) and school days missed. This school district requires HIPAA waivers to allow communication between school nurses and local health care providers and will be able to provide information on recorded school absences once informed consent forms are obtained. Secondary outcome measures include frequency of rescue inhaler use, as well as asthma exacerbations, outpatient clinic visits, and ED visits (see Table 2). Lung capacity will be obtained using spirometry pre- and postintervention [32] to obtain relevant lung function variables, such as FEV1 (forced expiratory volume in 1 second) and FEV1/FVC (forced vital capacity). Measures from the control group will include medication counts, number of asthma exacerbations, ED and outpatient clinic visits, and spirometry measures on enrollment, during the intervention phase, and again at the end of the intervention.

**Table 2:** Outcome measures and their details.

Outcome variable	Description	Source of data	Type of measure
Medication adherence—asthma medication ratio (primary outcome)	Medication administration data uploaded to the app	Parents	Continuous
Medication adherence—asthma medication ratio (primary outcome)	Medication counts performed monthly by the principal investigator	Principal investigator	Continuous
School days missed	School absences secondary to asthma symptoms	Nurses	Continuous
Asthma exacerbations	Number of asthma exacerbations uploaded to the app	Parents	Continuous
Frequency of rescue inhaler use	Albuterol usage for wheezing episodes	Parents	Continuous
Emergency department visits	Number and dates of emergency department visits uploaded to the app	Parents	Categorical
Outpatient clinic visits	Number and dates of outpatient clinic visits uploaded to the app	Parents	Continuous

#### Data Security

Biometric data are shared by the parent with the school nurse and PI through the AMD-Sp app by selecting and sending all uploaded information directly to the designated recipients. The app will be password protected to prevent unauthorized access to the information in case of phone loss [33].

#### Power Analysis

Formal power analyses are not presented for this study, because (1) we do not have useful effect size estimates and (2) the resources available for K23 research virtually insure an underpowered research design. Our sample of 20 (10 in each group) thus represents far less than .80 power. We will have power to detect an effect size of 1.16 in the mean asthma medication ratio between the intervention group and control group; the power to detect a large effect size (0.80) is .53 [34,35]. Our goals are to arrive at effect size estimates for future work.

#### Statistical Analysis

The main analysis for Aim 3 will be to compare the average adherence between the case and control groups. This comparison will be made via PROC REG in SAS, version 9.4 (SAS Institute) [36]. We will also compare mean rates of the asthma medication ratio using the Wilcoxon rank-sum test. A 1-sided *P* value of .05 will be considered significant for all analyses. I anticipate that the results will support the rationale for an RCT focused on a scalable, multisite, multicounty version of this intervention, with state-level stakeholders such as Select Health, at the conclusion of this study.

## Results

The University of South Carolina Institutional Review Board reviewed the study protocols for Aims 1 and 2 and these were approved after expedited review. The mobile app translation, initial usability testing, and app software refinement were completed in 2019. Analysis is in progress. Preliminary protocol testing is underway; we anticipate that the waiting-list RCT, using the refined protocol developed in Aim 2, will commence in fall 2020.

## Discussion

This collaborative mHealth intervention study addresses a gap in our current understanding of best practices for asthma management in specific vulnerable populations. Tailored, technology-based solutions have the potential to successfully address issues affecting asthma management, including communication barriers, accessibility issues, medication adherence, and suboptimal technological interventions.

## Abbreviations

AMD-Sp: Spanish-language version of AsthmaMD
ED: emergency department
FEV1: forced expiratory volume in 1 second
FVC: forced vital capacity
HIPAA: Health Insurance Portability and Accountability Act
LCSD: Lancaster County School District
mHealth: mobile health
NIH: National Institutes of Health
PCP: primary care provider
PI: principal investigator
RCT: randomized controlled trial
